# Lactic Acid Bacteria Strains Differently Modulate Gut Microbiota and Metabolic and Immunological Parameters in High-Fat Diet-Fed Mice

**DOI:** 10.3389/fnut.2021.718564

**Published:** 2021-09-09

**Authors:** Emanuel Fabersani, Antonela Marquez, Matías Russo, Romina Ross, Sebastián Torres, Cecilia Fontana, Edoardo Puglisi, Roxana Medina, Paola Gauffin-Cano

**Affiliations:** ^1^Facultad de Agronomía y Zootecnia, Universidad Nacional de Tucumán, Tucumán, Argentina; ^2^Centro de Referencia para Lactobacilos -CONICET, Tucumán, Argentina; ^3^Instituto de Biotecnología Farmacéutica y Alimentaria -CONICET, Tucumán, Argentina; ^4^Facultad Ciencias de la Salud, Universidad del Norte Santo Tomás de Aquino, Tucumán, Argentina; ^5^Instituto de Bioprospección y Fisiología Vegetal -CONICET, Tucumán, Argentina; ^6^Department for Sustainable Food Process, Università Cattolica del Sacro Cuore, Piacenza, Italy

**Keywords:** lactobacilli, probiotic, adipocytes, macrophages, high-fat diet-fed mice, gut microbiota

## Abstract

**Background:** Dietary strategies, including the use of probiotics as preventive agents that modulate the gut microbiota and regulate the function of adipose tissue, are suitable tools for the prevention or amelioration of obesity and its comorbidities. We aimed to evaluate the effect of lactic acid bacteria (LAB) with different adipo- and immuno-modulatory capacities on metabolic and immunological parameters and intestinal composition microbiota in high-fat-diet-induced in mice fed a high-fat diet

**Methods:** Balb/c weaning male mice were fed a standard (SD) or high-fat diet (HFD) with or without supplementation with *Limosilactobacillus fermentum* CRL1446 (CRL1446)*, Lactococcus lactis* CRL1434 (CRL1434), or *Lacticaseibacillus casei* CRL431 (CRL431) for 45 days. Biochemical and immunological parameters, white-adipose tissue histology, gut microbiota composition, and *ex vivo* cellular functionality (adipocytes and macrophages) were evaluated in SD and HFD mice.

**Results:** CRL1446 and CRL1434 administration, unlike CRL431, induced significant changes in the body and adipose tissue weights and the size of adipocytes. Also, these strains caused a decrease in plasmatic glucose, cholesterol, triglycerides, leptin, TNF-α, IL-6 levels, and an increase of IL-10. The CRL1446 and CRL1434 obese adipocyte in *ex vivo* functionality assays showed, after LPS stimulus, a reduction in leptin secretion compared to obese control, while with CRL431, no change was observed. In macrophages from obese mice fed with CRL1446 and CRL1434, after LPS stimulus, lower levels of MCP-1, TNF-α, IL-6 compared to obese control were observed. In contrast, CRL431 did not induce modification of cytokine values. Regarding gut microbiota, all strain administration caused a decrease in Firmicutes/Bacteroidetes index and diversity. As well as, related to genus results, all strains increased, mainly the genera *Alistipes, Dorea, Barnesiella*, and *Clostridium* XIVa. CRL1446 induced a higher increase in the *Lactobacillus* genus during the study period.

**Conclusions:** The tested probiotic strains differentially modulated the intestinal microbiota and metabolic/immunological parameters in high-fat-diet-induced obese mice. These results suggest that CRL1446 and CRL1434 strains could be used as adjuvant probiotics strains for nutritional treatment to obesity and overweight. At the same time, the CRL431 strain could be more beneficial in pathologies that require regulation of the immune system.

## Introduction

Until the beginning of the 20th century, obesity was considered an individual problem caused by uncontrolled eating and personal responsibility. Currently, obesity is described as an immuno-metabolic, heterogeneous, polygenic, and multifactorial disease. It is characterized by excess and dysfunction of the adipose tissue and alteration of the gut microbiota, affecting human health. This multifactorial epidemic represents the main problem for public health worldwide (1).

So far, despite significant research, the underlying mechanisms involved in the development of obesity have not been fully elucidated. However, it has been established that three main factors: diet, genetics, and intestinal microbiota, interact with each other and set the immuno-metabolic profile of an individual. The intestine, brain, and adipose tissue are the principal organs involved in developing this disease.

The gut microbiota has been linked to diet, and it is widely recognized that its composition conditions an individual's metabolic profile. Studies have shown that dysbiosis and low species diversity are associated with obesity (2). Besides, the immune system also plays a crucial role in regulating gut microbiota, whereby immunity, further their participation in infection/inflammation activities, is fundamental to maintaining a healthy state or developing metabolic disease (3).

The human gut microbiota encompasses a densely populated ecosystem that provides essential functions for the host, affecting the metabolism and immune maturation in a state of symbiosis. Gut microbiota composition changes leading to dysbiosis state could propitiate the development of obesity; in fact, studies in animals have suggested that dysbiosis is a causal agent of this disease (4).

In humans and mice, obesity correlates, in most cases, with changes in the relative abundance of two dominant bacterial *phyla* in the intestine: Bacteroidetes and Firmicutes. It was reported an increase of the Firmicutes and a decrease in Bacteroidetes, which increases the Firmicutes/Bacteroidetes (F/B) index (5). Also, transfer the gut microbiota from genetically obese (*ob/ob*) mice to germ-free wild-type (WT) mice have been shown to lead to an increase in fat mass, conducting the speculation that the intestinal microbiota promotes obesity by increasing the host's ability to draw energy from ingested foods, and controlling food intake and energy distribution (6).

The inflammation of adipose tissue (AT) was proposed as a central mechanism in developing obesity and its associated metabolic disorders (7, 8). Immune cells resident into AT are the second major cellular component after adipocytes and, as such, play an essential role in maintaining local and systemic homeostasis. Obesity-induced changes in cell numbers, functionality, and adipokine profile result in the activation of the local and systemic inflammatory response, marking the transition from a simple increase of adiposity to insulin resistance, diabetes mellitus type II (DM2), or non-alcoholic fatty liver (NAFLD) associated with obesity (7). Obesity induces low-grade inflammation or “meta-inflammation” (inflammation of metabolic organs) throughout the body. This chronic state of inflammation is mediated by macrophages located within the colon, liver, muscle, and adipose tissue (9).

Consequently, immunity plays a fundamental role in health or metabolic disease, supporting the alternative strategies for treating these metabolic diseases using immunobiotics (immunoregulatory probiotics or microbial strains able to regulate the mucosal immune system beneficially). Effectively, several authors have investigated probiotics for their potential use to treat or prevent nutritional disorders (5, 10–12). These beneficial strains influence the regulation of the cytokine profile, adipokines, and intestinal microbiota structure and ameliorate diet-induced immuno-metabolic alterations (13).

Establishing a process of selection of strains with probiotic potential for obesity represents a great challenge, taking into account the wide range by which a bacterium could exert its beneficial effect. We focused our study on the premise that the meta-inflammation associated with obesity is responsible for developing this disease and taking leptin as the central adipokine in this process. We previously selected non-inflammatory lactic acid bacteria (LAB) strains with the capacity to modulate adipokine secretion in adipocyte cultures (13). Our *in vitro* previous results allowed us to establish the capacities of different LAB to modulate the immune system or adipose tissue, and we selected three strains with specific characteristics to be studied in the following *in vivo* essay*: Limosilactobacillus fermentum* CRL1446*, Lactococcus lactis* CRL1434, or *Lacticaseibacillus casei* CRL431.

In this work, we aimed to evaluate the effect of LAB with different adipo- and immuno-modulatory capacities on metabolic and immunological parameters and the composition of intestinal microbiota in a high-fat diet-fed mice model.

## Materials and Methods

### Bacterial Strains and Culture Conditions

The LAB used in this study were obtained from the culture collection of the Reference Center for Lactobacillus (CERELA)-CONICET, Tucumán, Argentina. *Limosilactobacillus fermentum* CRL1446 (CRL1446)*, Lactococcus lactis* CRL1434 (CRL1434), or *Lacticaseibacillus casei* CRL431 (CRL431) were selected in previous studies by their non-inflammatory properties and capacities to modulate adipokine secretion (leptin) in cells culture (13). The LAB were cultured in Man-Rogosa-Sharpe (MRS) broth (MRS, Britania, Buenos Aires, Argentina) for 16 h at 37 ° C (late exponential phase), following the methodology previously described by Fabersani et al. (13).

### Animals, Diets, and Experimental Design

Balb/c male mice aged 21 days (*n* = 80) were obtained from the closed random-bred colony maintained at CERELA. Experiments were carried out following the Guide for the Care and Use of Laboratory Animals Center of CERELA. The study protocol was approved by the Ethical Committee (approval number CRL-BIOT- EF-2012 / 2A).

In the adaptation period, the mice were housed in acclimated cages at 22 ± 2 ° C, with a light / dark cycle of 12 h, and fed a standard diet (SD). After that, some animals were switched to the high-fat diet (HFD). The animals were randomly separated into the following groups (*n* = 16 each group): **SD:** mice fed standard diet; **HFD:** mice fed high-fat diet; **HFD-CRL1446:** mice fed HFD supplemented with CRL1446; **HFD-CRL1434:** mice fed HFD supplemented with CRL1434 and **HFD-CRL431:** mice fed HFD supplemented with CRL431. The HFD provided 20% kcal as protein, 20% kcal as carbohydrate, and 60% kcal as fat (energy density: 5.21 kcal/g diet) (Research Diets Inc., D12492, Rodent Diet with 60 kcal% Fat, NJ, USA). The SD provided 20% kcal as protein, 60% kcal as carbohydrate, and 20% kcal as fat (energy density: 3.21 kcal/g diet) (Association of Argentine Cooperatives, Buenos Aires, Argentina). Mice had free access to diets for 45 days. The mice received a daily probiotic dose of each LAB in the drinking water (1.0 × 10^8^ CFU ml^−1^) (14–16). Each animal drinks about 1–4 ml per day, receiving between 1.0 × 10^8^ and 4.0 × 10^8^ CFU of probiotic LAB per day. The drinking water supplemented with LAB was replaced every 12 h.

Body weight was measured weekly, and caloric intake was accounted for from daily food consumption. At the end of the study, animals fasted for 12 h and subsequently anesthetized (intraperitoneally into the right lower quadrant of the abdomen) with 30 μl of the anesthetic mixture containing 2 mg ketamine and 0.2 mg xylazine (independently of the bodyweight), and sacrificed by cervical dislocation. Blood samples were collected in tubes containing EDTA, centrifuged to obtain plasma, and was kept at −20°C to analyze metabolic parameters. The fecal contents were collected at the end of the experimental period (45 days) for gut microbiota analyses. Fat pads of epidydimal and mesenteric adipose tissues (total adipose tissue; TAT) were removed and rinsed thoroughly with ice-cold saline (PBS), weighed, and fixed in a 10% neutral formalin buffered solution for histological analysis.

### Analysis of Metabolic and Inflammatory Parameters

Triglycerides, total cholesterol, and glucose were analyzed in the plasma samples. The analysis was carried out by enzymatic methods using commercial kits (Wiener Lab, Rosario, Argentina). Also, plasmatic leptin levels were determined by enzyme-linked immunosorbent assay (Mouse / Rat Leptin Quantikine ELISA Kit, MN, USA). Plasma insulin levels were measured using ELISA (Mouse Insulin ELISA Kit, Alpco Diagnostics, USA). The chemokine monocyte chemoattractant protein- 1 (MCP-1) and the cytokines, tumor necrosis factor-alpha (TNF-α), interleukin (IL)-6 (IL-6), and IL-10 were determined by flow cytometry using the BD™ Cytometric Bead Array (CBA) Mouse Inflammation Kit (BD Bioscience, 560485, San Diego, CA, USA).

### Histological Analysis of Adipocytes

Paraffin-embedded epididymal adipose tissues were sectioned to a thickness of 4–5 μm and fixed to glass slides. Slides were deparaffinized and stained with hematoxylin-eosin. The adipocyte number and sizes of each group were determined according to the methodology described by Gauffin-Cano et al. (10). For the histological analysis was used the Carl Zeiss-Axio Vision software, Release 4.8. Adipocyte numbers and sizes were measured in 100 cells of two sections of epididymal adipose tissue per mouse (*n* = 8 each group). Adipocyte sizes were expressed as area ranges using the following ranges: areas <500 (1); 500–1000 (2); 1,000–2,000 (3); 2,000–4,000 (4) and >4,000 (5) μm2) to be able to compare between the different groups.

### *Ex vivo* Functionality Evaluation of Peritoneal Macrophages and Adipocytes

*Ex vivo* functionality studies were carried out on day 45 of the experiment. Macrophages cells were collected from the peritoneal cavity, according to Fabersani et al. (16) in 5 mL of cold Dulbecco's Modified Eagles Medium (DMEM) (Gibco, #41965-039, CA, USA), containing 10% inactivated fetal bovine serum (FBS) (Gibco, 10082139, CA, USA), streptomycin/penicillin (Sigma ALDRICH P 4333, MO, USA). The isolated macrophages were plated into the flask (Costar® 24, NY, USA) at a concentration of 10^6^ cells/mL in DMEM and incubated for 2 h at 37°C in an atmosphere containing 5% CO_2_. Then non-adhered cells were washed out with phosphate-buffered saline (PBS) (135 mM NaCl, 8.1 mM Na_2_HPO_4_, 1.5 mM KH_2_PO_4_, and 3.0 mM KCl, pH 7.4). To evaluate the functionality of peritoneal macrophages (from each mice group), adhered macrophages were cultured overnight into 24-well flat-bottom polystyrene microtiter plates (Costar® 24 well flat, #3524, NY, USA) at the concentration of 1 x 10^5^ cells/mL in DMEN. The culture media were changed before LAB stimulation, and then macrophages were incubated in the presence of 100 μL of a cell suspension 11 × 0^7^ CFU/ml of each strain for 24 h. For positive stimulation control, macrophages were incubated in the presence of purified lipopolysaccharide (LPS) from *E. coli* serotype O26: B6 (SIGMA-ALDRICH, # L2654, MO, USA) at a concentration of 1 μg/mL. Non-stimulated peritoneal macrophages were evaluated as controls of basal cytokine production. Cell viability was determined with Trypan-blue (SIGMA-ALDRICH, # T6146, MO, USA). The cell culture supernatants were collected and stored at −20°C until cytokine determination. Secreted TNF-α, IL-6, IL-10, and MCP-1 by macrophages were quantified in cell supernatants using BD™ Cytometric Bead Array (CBA) Mouse Inflammation Kit (BD Bioscience, 560485, San Diego, CA, USA).

To study the functionality of adipocytes from the different groups, adipocytes from epididymal adipose tissue were isolated by collagenase digestion (Collagenase from *Clostridium histolyticum* Type II #C6885 SIGMA, USA.) as was previously described (16). Briefly, adipose tissues were digested for 1 h at 37°C in DMEM high glucose containing 1.5 mg/mL collagenase II and supplemented with 10% (v/v) penicillin/streptomycin and FBS. The suspension was centrifuged (400 rpm for 5 min). The stromal-vascular cells (capillary, endothelial, mast, macrophage, and epithelial cells) in the bottom were removed by aspiration, and the mature adipocytes were washed with supplemented DMEM high glucose. Isolate adipocyte cells were inoculated in 0.5 mL of plating medium into 24-well plates at a concentration of 5 x 10^5^ cells/wells. The cells were maintained in a humidified 5% CO_2_ atmosphere for 24 h at 37°C. The medium was changed before the stimulation, and then, adipocyte cells were incubated with 100 μL of a cell suspension of 11 × 0^7^ CFU/mL of each strain for 24 h. The positive stimulation control were adipocytes from different mice groups incubated in the presence of purified LPS from *E. coli* serotype O26: B6 at a concentration of 1 μg/mL. Non-stimulated adipocytes were evaluated as controls of basal leptin production. Leptin concentrations secreted by adipocytes were measured in cell supernatants with a DuoSet kit (R&D Systems, Minneapolis, MN, USA). The assays were performed according to the manufacturer's instructions.

Each parameter was determined in triplicate in two independent experiments.

### DNA Extraction and High-Throughput Sequencing of 16S rRNA Gene Amplicons

Fecal samples from each group (*n* = 3) were collected at 20 and 45 days of study in sterile Eppendorf tubes and stored at −20 °C until use. The DNA extraction was performed using a specific commercial kit for fecal samples (QIAamp DNA Stool Mini Kit, Hilden, Germany). The V3-V4 bacterial regions of the 16S rRNA were amplified by PCR using primer pairs, 343F (5′-TACGGRAGGCAGCAG-3′) and 802R (5′-TACNVGGGTWTCTAATCC-3′), according to Polka et al. (17). The PCR product set was sent to Fasteris SA (Geneva, Switzerland) for sequencing using the TruSeq ™ DNA Sample Preparation Kit (Illumina Inc., San Diego, CA, USA). High throughput sequencing was performed on a MiSeq Illumina instrument (Illumina Inc.) using the V2 chemistry in a 2 x 250 configuration to cover the entire amplicon length, estimated at ~450 bp. The matched end fitting and quality filtering were performed using Flash software (18). Sample multiplexing was carried out using information from the barcoding DNA samples and the Mothur platform. Chimera elimination was performed using the UCHIME algorithm (19). Then, the sequences obtained were grouped according to their similarity in different operational taxonomic units (OTUs) and annotated at taxonomic levels, comparing them with the public reference database SILVA (20).

Diversity indexes were calculated using the Mothur platform using default parameters and the mean method in the clustering step (21). Diverse wealth-measuring diversity parameters such as Sobs or Chao as well as diversity in terms of homogeneity, such as Shannon and Simpson, were calculated from operational taxonomic units (OTUs) grouped by 97% and using a standardized subset of 23,000 sequences Selected at random, after multiple blends (10,000X) from the original database. At the phylum and genus level, the taxonomic assignment of the subset of high-quality readings was performed using the Bayesian RDP classifier (22), using a cutoff confidence assignment of 0.8. Beta diversity was analyzed by performing the Unifrac-weighted analysis.

### Statistical Analysis

Statistical analyses were performed using SPSS version 17.0 software (SPSS Inc., Chicago, IL, USA). Significant differences were determined by applying one-way ANOVA with *post hoc* Tukey's test or non-parametric Kruskal–Wallis test with Dunn's multiple comparisons test. In every case, *P* < 0.05 were considered statistically significant.

## Results

### CRL1446 and CRL1434 Administration Induced a Decrease in Body Weight Gain and the Feed Conversion Ratio in HFD Mice

**Figure 1:**
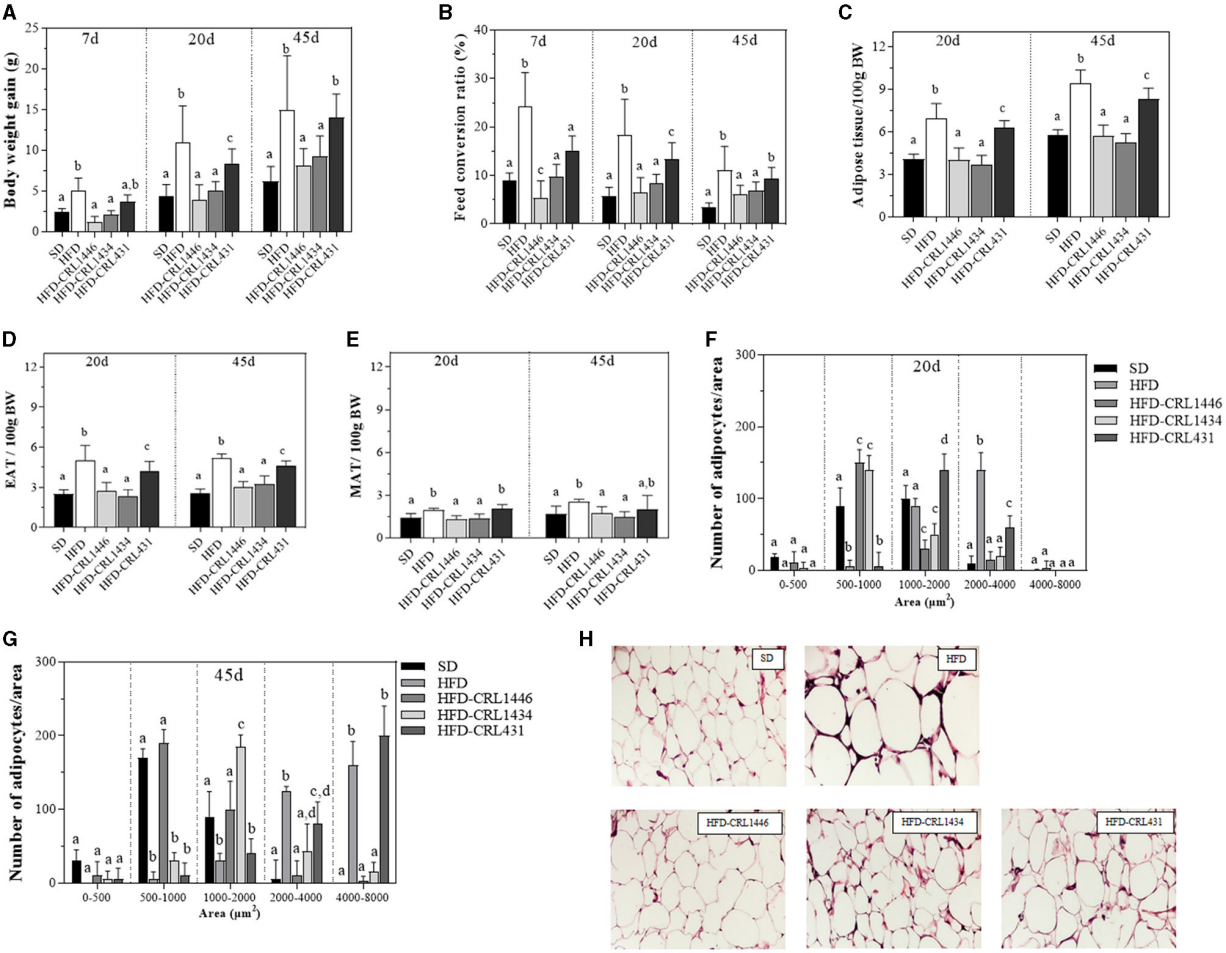
Body weight gain **(A)** and Feed conversion ratio (FCR) **(B)** at days 7, 20, and 45, Relative weight of epididymal adipose tissue (EAT) **(C)**, Mesenteric adipose tissue (MAT) **(D)**, and Total adipose tissue (TAT) **(E)** at days 20 and 45, Distribution of adipocyte size at day 20 **(F)**, Distribution of adipocyte size at day 45 **(G)**, and Histology of epididymal adipose tissue at day 45 **(H)** of mice under the administration of standard Diet (SD), or a high-fat diet (HFD) with administration of CRL1446 (HFD-CRL1446), CRL1434 (HFD-CRL1434), and CRL431 (HFD-CRL431) at a dose of 1 x 10^8^ CFU/mL. Control mice were fed with a SD or HFD without bacterial supplementation. The FCR is the relation between the food consumed (g) and the body weight gain (g) expressed as a percentage. Adipocyte cell sizes are expressed as area ranges: <500, 500–1,000, 1,000–2,000, and 2,000–4,000 μm^2^. Photomicrographs 40X of representative HE-stained slides of AT are only shown on day 45. Each group of data (*n* = 6) is represented by the mean ± SEM (standard error mean). In Figure **(A)** and **(B)**, in the same period of treatment (7, 20, or 45 d), data with different lowercase letters (a–c) are significantly different (*P* < 0.05). In Figure **(C), (D)**, and **(E)**, in the same period of treatment (20 or 45 d), data with different lowercase letters are significantly different (*P* < 0.05). In Figure **(F)** and **(G)**, data with different lowercase letters in the same adipocyte cell sizes (area ranges) are significantly different (*P* < 0.05). These evaluations were carried out using the ANOVA statistical analysis.

Figure 1 shows the effect of different dietary treatments on body weight gain (BWG) **(A)** and the feed conversion ratio (FCR) (B) at 7, 20, and 45 days of bacterial treatment. Body weight gain (BWG) is defined as the body weight increase in a determined time (Final body weight—Initial body weight). We observe that the HFD induces an increase of twice the BWG at day 45, concerning the SD group. On day 45, the groups HFD-CRL1446 (*P* = 0.0025) and HFD-CRL1434 (*P* = 0.0175) showed a significant reduction of BWG compared with the HFD group in all studied periods (Figure 1A). On day 45, the HFD supplementation with strain CRL431 not showed significant changes in BWG compared to the HFD group (Figure 1A).

The food intake and BWG allow evaluating the capacity to transform grams of food consumed into grams of body weight. This is defined as feed conversion ratio (FCR) [Body weight gain (g)/Food consumed (g) x 100]. The FCR is closely related to the structure of the intestinal microbiota and diet. The modification of the intestinal ecosystem by the administration of LAB may influence this parameter. The FCR was determined on days 7, 20, and 45 (Figure 1B). The HFD showed a significant increase (63–69%) of the FCR compared to the SD group at all times evaluated (*P* < 0.0001). Only the HFD supplementation with the strains CRL1446 and CRL1434 induced a significant (*P* = 0.0018 and *P* = 0.0076, respectively) reduction in FCR in all the assays (45 and 38%, respectively at 45 days) (Figure 1B).

### The Adipose Tissue Weight and Adipocyte Area Were Reduced by CRL1446 and CRL1434 Administration in HFD Mice

We evaluated the weight of total adipose tissue (TAT) (Figure 1E), epididymal adipose tissue (EAT) (Figure 1C), and mesenteric adipose tissue (MAT) (Figure 1D). The HFD significantly (*P* < 0.0001) increased TAT weight by ≈1.69 and 1.65 fold at days 20 and 45 of treatment concerning the SD group. In addition, we observed that this effect was more significant in the EAT than MAT compared with the SD group (Figures 1C,D, respectively). At days 20 and 45, the supplementation of HFD with CRL1446 and CRL1434 strains induced a significant decrease in all adipose tissue (TAT, EAT, and MAT), reaching SD values. CRL431 strain induced a significant reduction of TAT and EAT weight without reach the SD values.

In histological sections of EAT, we analyzed changes in the adipose tissue structure (Figures 1F,G), determining the number of adipocytes with different area sizes (adipocytes with areas sizes between 0–500; 500–1,000; 1,000–2,000; 2,000–4,000 and >4,000 μm^2^). On day 20 of treatment (Figure 1F), HFD significantly induced the increase of the hypertrophied adipocytes number (area between 2,000 and 4,000 μm^2^) and reduced the adipocyte number between 500 and 1,000.

In the HFD group, at 45 days (Figure 1G), the more significant adipocyte number with an area between 3,000 and 8,000 μm^2^ was observed. Furthermore, at 20 or 45 days of treatment, we observed fewer small adipocytes (area between 1,000 and 2,000 μm2) in HFD mice than in SD mice. At 20 days, supplementation of HFD with strains CRL1446 and CRL1434 induced a significant reduction of medium (1,000–2,000 μm2) and large (2,000–4000 μm^2^) adipocytes, while small adipocytes (500–1,000 μm2) significantly increased compared to the HFD group (Figures 1F,G). The CRL431 strain increased median adipocytes (1,000–2000 μm^2^) and significantly reduced large adipocytes (2,000–4,000 μm^2^); this effect was lower than the other strains (CRL1446 and CRL1434). At 45 days (Figure 1G), only CRL1446 maintained increasing the small adipocytes (1,000–2,000 μm^2^) with an almost total reduction of hypertrophied adipocytes (≥3,000 μm^2^). In Figure 1H we showed, representative images of hematoxylin- and eosin-stained sections of EAT in mice fed HFD with and without administration of CRL1446 (HFD-CRL1446), CRL1434 (HFD-CRL1434), and CRL431 (HFD-CRL431) at 45 days.

### Plasma Glucose, Triglyceride, Cholesterol, and Insulin and Leptin Levels Were Reduced by CRL1446 and CRL1434 Administration in HFD Mice

**Figure 2:**
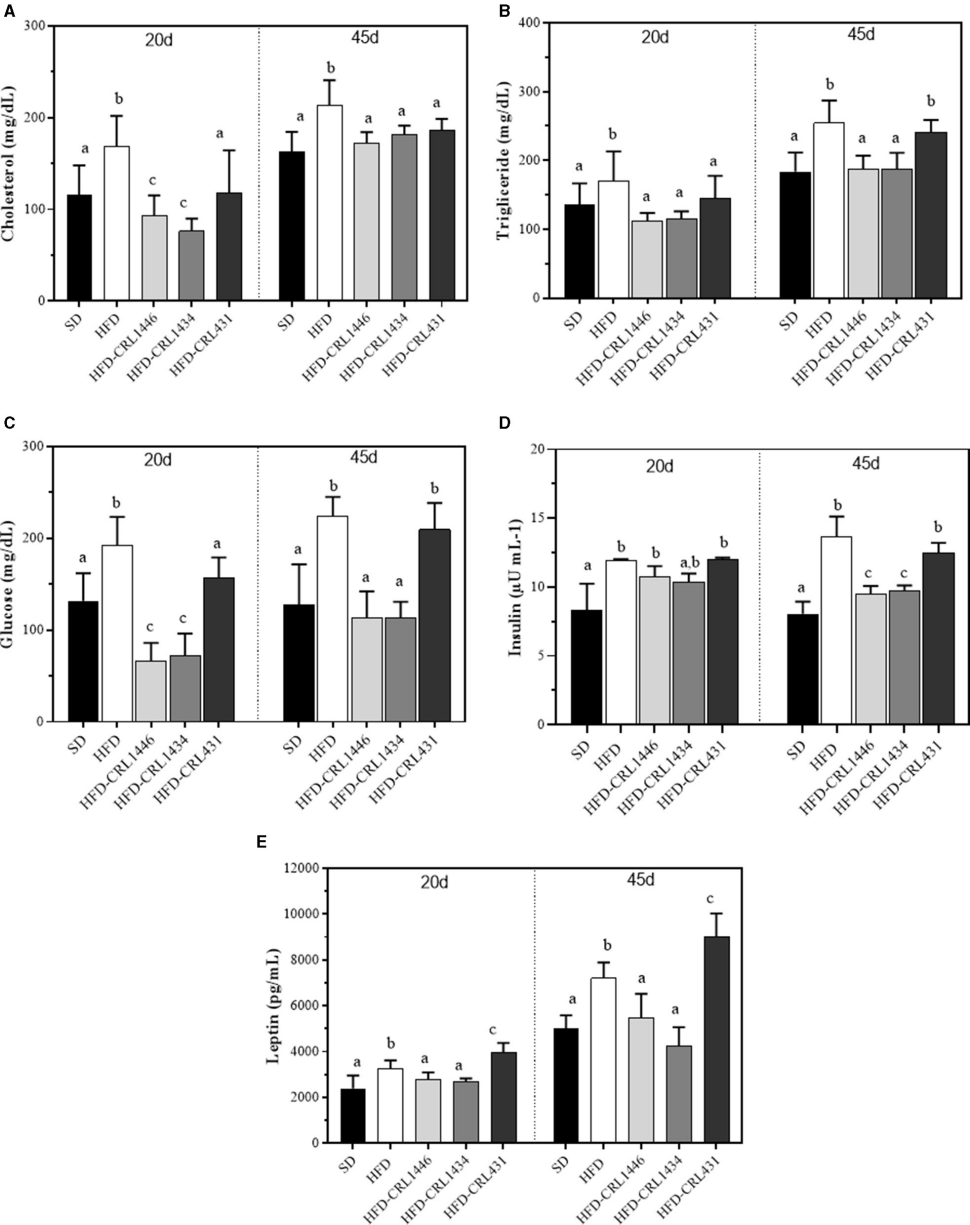
Plasma levels of glucose **(A)**, triglycerides **(B)**, total cholesterol **(C)**, and leptin **(D)** at 20 and 45 days of standard diet (SD) or a high-fat diet (HFD) administration with and without supplementation of CRL1446 (HFD-CRL1446), CRL1434 (HFD-CRL1434), and CRL431 (HFD-CRL431) at a dose of 1 x 10^8^ CFU/mL. Controls mice were fed with SD or HFD without bacterial supplementation. Each group of data (*n* = 6) represents the mean ± SE (standard error). Data with different lowercase letters in the same period of treatment (20 or 45 d) are significantly different (*P* < 0.05) according to the ANOVA test.

We analyzed the effects of HFD and LAB administration in glucose, triglycerides, total cholesterol, and leptin levels for 45 days (Figures 2A–D). Our results showed strain-dependent effects on carbohydrates and lipids metabolism. Related to glucose and triglyceride levels, the HFD supplementation with CRL1446 and CRL1434 significantly reduces these markers compared to the HFD group (Figures 2A,B, respectively). While that administration of CRL431 only significantly decreased glucose and triglyceride levels at 20 days. Regarding the total cholesterol levels (Figure 2C), the supplementation with CRL1446 and CRL1434 induced a decrease compared to the HFD group at 20 and 45 days. CRL431 induced significant changes in total cholesterol only at 45 days of administration.

Insulin levels (Figure 2D) in HFD group are significantly increased compared to SD group. the HFD supplementation with CRL1446 and CRL1434 induced a significant decrease concerning HFD mice, whereas in HFD-CRL431 group, no change was observed

In Figure 2E, we observed that HFD had an adipose-modulatory effect since significantly increased plasma leptin levels (1.37 times at 20 days and 1.44 times at 45 days) than SD. The administration of CRL431 strain induced an increase in leptin levels, which was significantly higher than HFD. The CRL1446 and CRL1434 have the opposite effect; these strains induced a significant reduction of leptin levels, reaching the levels of the SD group.

### Inflammatory Status in HFD Mice Was Modulated by LAB in a Strain-Dependent Way

**Figure 3:**
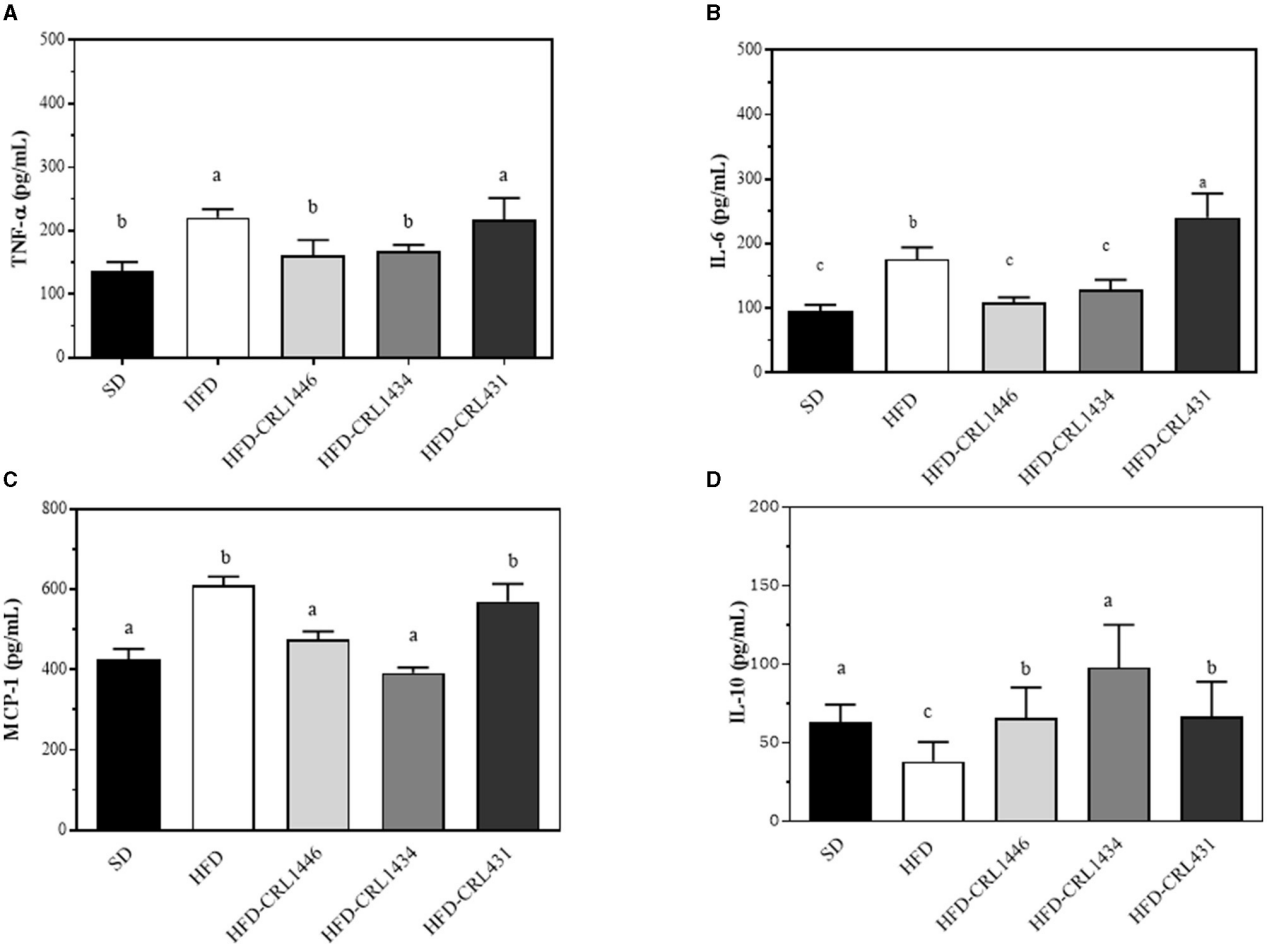
**(A–D)** Levels of plasma pro-inflammatory (IL-6, TNF-α and anti-inflammatory (IL-10) cytokines and pro-inflammatory (MCP-Q22 1) chemokine at 45 d after administration of the standard Diet (SD), or the high-fat diet (HFD), with and without administration of CRL1446 (HFD-CRL1446), CRL1434 (HFD-CRL1434), and CRL431 (HFD-CRL431) at a dose of 1 x 10^8^ CFU/mL. Each data group (*n* = 6) is represented by the mean ± SEM (standard error mean). Data with different lowercase letters are significantly different (*P* < 0.05) according to the ANOVA test.

We determined plasma levels of cytokines (TNF-α, IL-6, and IL-10) and the chemokine MCP-1 at 45 days, as shown in Figure 3. HFD, compared to SD, significantly increased TNF-α, IL-6, and MCP-1 (Figures 3A–C), while IL-10 was reduced, demonstrating a pro-inflammatory effect of diet (Figure 3D). Supplementation with CRL1446 and CRL1434 strains completely restored pro-inflammatories molecules (IL-6, TNF-α y MCP-1), achieving the levels of the SD group. Regarding IL-10 levels, this was partially increased with CRL1446 and CRL431, compared with SD group. At the same time, CRL1434 administration recovered this cytokine close to SD levels. Supplementation with strain CRL431 induced a significant increase in IL-6 levels compared to the HFD group.

### CRL1446, CRL1434, and CRL431 Showed Different Influence in Adipocyte and Macrophage Functionality in HFD Mice

We further evaluated the *ex vivo* functionality of peritoneal macrophages by producing TNF-α, IL-6 (pro-inflammatory molecules), and IL-10 (anti-inflammatory molecule) cytokines, and MCP-1 chemokine (pro-inflammatory molecule) (Table 1). We did not observe significant differences in the basal production of the cytokines (Control, supernatant of culture cells without stimulus) of the different groups, except for TNF-α at 45 days and MCP-1compared with SD mice. When macrophages were stimulated with LPS, HFD reduced IL-10 production at 20 and 45 days and increased TNF-α, IL-6, and MCP-1 at 45 days, concerning the macrophages of the SD group. The LAB administration to macrophages challenged with LPS restored IL-10 levels to values similar to the SD group. The CRL1434 strain induced the highest levels of IL-10. On the other hand, the administration of CRL1446 and CRL1434 produced a significant reduction in the levels of pro-inflammatory cytokines, and chemokines (TNF-α, IL-6, and MCP-1) increased by HFD. The CRL431 strain did not induce a significant change in these pro-inflammatory molecules in the HFD group.

**Table 1:** TNF-α, IL-6, MCP-1, and IL-10 *ex vivo* production by LPS-stimulated or non-stimulated peritoneal macrophages at 20 and 45 days.

**Period**	**Cytokines**	**Stimulus**	Cytokines from peritoneal macrophage (pg/mL)^*^				
			**SD**	**HFD**	**HFD-CRL1446**	**HFD-CRL1434**	**HFD-CRL431**
20 d	TNF-α	Control	29.4 ± 4.5^a^	34.4 ± 6.3^a^	28.0 ± 3.3^a^	38.5 ± 3.2^a^	37.1 ± 3.3^a^
		LPS	1087.1 ± 63.2^c^	5866.1 ± 337.8^a^	1482.5 ± 128.6^b^	2674.8 ± 240.5^ab^	4036.8 ± 500.5^c^
	IL-6	Control	16.6 ± 4.1^a^	29.8 ± 5.9^a^	19.9 ± 3.1^a^	27.1 ± 5.3^a^	27.3 ± 4.4^a^
		LPS	520.9 ± 63.3^b^	2206.5 ± 512.6^a^	1090.8 ± 164.3^b^	1128.6 ± 78.9^b^	1609.3 ± 275.2^b^
	MCP-1	Control	6889.7 ± 394.5^a^	7472.5 ± 481.6^a^	5834.2 ± 672.9^a^	5676.5 ± 653.0^a^	5960.6 ± 529.9^a^
		LPS	17468.1 ± 877.5^b^	19966.9 ± 1259.9^a^	14285.3 ± 3187.4^b^	14788.2 ± 2928.0^b^	15581.0 ± 3106.5^b^
	IL-10	Control	3.7 ± 0.8^a^	1.3 ± 1.2^a^	2.5 ± 1.8^a^	5.4 ± 3.5	3.7 ± 2.3^a^
		LPS	37.0 ± 5.3^b^	8.4 ± 1.3^c^	29.5 ± 2.9^b^	51.8 ± 7.8^a^	33.9 ± 3.8^b^
45 d	TNF-α	Control	735.8 ± 144.1^a^	1788.8 ± 316.0^a^	632.3 ± 176.3^a^	680.8 ± 82.4^a^	1013.8 ± 108.7^a^
		LPS	5194.8 ± 912.9^b^	25365.3 ± 2233.3^a^	3365.8 ± 373.2^b^	7224.0 ± 816.5^b^	16447.8 ± 3099.0^a^
	IL-6	Control	41.7 ± 4.4^a^	59.8 ± 12.6^a^	41.7 ± 4.4^a^	55.3 ± 8.4^a^	64.6 ± 10.7^a^
		LPS	1922.3 ± 110.2^b^	6802.5 ± 1247.0^a^	2224.0 ± 359.3^b^	3036.3 ± 598.2^b^	5926.5 ± 1394.8^a^
	MCP-1	Control	13086.1 ± 1025.1^a^	17858.7 ± 1048.5^a^	14175.2 ± 514.8^a^	14008.7 ± 662.8^a^	18790.8 ± 1715.9^a^
		LPS	16622.2 ± 1496.9^b^	28010.6 ± 1070.8^a^	18661.0 ± 771.1^b^	20542.3 ± 1886.5^b^	28737.6 ± 1242.8^a^
	IL-10	Control	5.1 ± 0.3^a^	2.3 ± 1.1^a^	2.3 ± 1.3^a^	6.5 ± 0.8^a^	6.8 ± 0.6^a^
		LPS	38.3 ± 2.6^a^	3.9 ± 1.4^c^	23.3 ± 2.5^b^	40.5 ± 3.0^a^	25.0 ± 3.7^b^

* *Peritoneal macrophage from mice under the standard diet (SD) or the high-fat diet (HFD) administration with and without administration of CRL1446 (HFD-CRL1446), CRL1434 (HFD-CRL1434), and CRL431 (HFD-CRL431) at a dose of 1 x 10^8^ CFU/mL. Peritoneal macrophages without stimulus were evaluated as controls of basal cytokine production (Control). Each group of data (n = 6) is represented by the mean ± SEM (standard error mean).*

*Different letters in some rows indicate significant differences. These evaluations were carried out using the ANOVA statistical analysis*.

The *ex-vivo* adipocyte functionality in obese mice treated with and without LAB administration was evaluated through leptin production (Table 2). Adipocytes from different groups showed a differential profile of leptin production. HFD did not affect the basal production (Control, the supernatant of epididymal adipocytes without stimulus) of this hormone concerning the SD group. Nevertheless, the HFD increased the response to the LPS stimulus, showing higher levels of leptin production in both times (20 and 45 days). Supplementation of HFD diet with LAB reduced leptin levels in LPS-stimulated adipocytes, and this reduction was significant when the diet was supplemented with CRL1446 and CRL1434, compared to the HFD group, whereas strain CRL431 did not show significant differences.

**Table 2:** Leptin production *ex vivo* production by LPS-stimulated or non-stimulated adipocyte cells at 20 and 45 days.

**Period**	**Stimulus**	Leptin from adipocyte (pg/mL)^*^				
		**SD**	**HFD**	**HFD-CRL1446**	**HFD-CRL1434**	**HFD-CRL431**
20 d	Control	12609 ± 5^a^	14131 ± 16^a^	16452 ± 92^a^	17203 ± 28^a^	15952 ± 59^a^
	LPS	16463 ± 23^a^	26733 ± 81^b^	18713 ± 36^a^	18885 ± 50^a^	22953 ± 52^a, b^
45 d	Control	12949 ± 8.14^a^	15501 ± 27^a^	10924 ± 4^a^	10697 ± 3^a^	14935 ± 5^a^
	LPS	28721 ± 87.51^a^	44763 ± 06^b^	24241 ± 40^c^	21651 ± 96^c^	36092 ± 30^c^

* *Adipocyte cells from mice under the standard diet (SD) or the high-fat diet (HFD) administration with and without administration of CRL1446 (HFD-CRL1446), CRL1434 (HFD-CRL1434), and CRL431 (HFD-CRL431) at a dose of 1 x 10^8^ CFU/mL. The cells were stimulated with lipopolysaccharide (LPS). Adipocyte cells without stimulus were evaluated as controls of basal cytokine production (Control). The mean ± SEM represents each group of data (n = 6). Different letters in the same rows indicate significant differences. These evaluations were carried out using the ANOVA statistical analysis*.

### Effect of HFD and LAB on the Gut Microbiota Composition

The microbial communities present in the fecal content samples from mice from the different groups were analyzed by massive and parallel sequencing of 16S rDNA amplicons. Table 3 (supplementary material) shows the alpha diversity analysis (Sobs, Chao, Shannon, and Simpson) and are calculated based on OTU species and abundance. The Sobs, Chao, and Ace (indexes of the species richness) showed that HFD induced a considerable reduction of theoretical intestinal microbial species compared with SD and LAB supplemented-HFD groups. The Shannon and Simpson indexes reflect community diversity (including species richness and species evenness). Interestingly, the HFD seems to maintain species diversity in terms of the evenness of the community because the index remained constant throughout the study period (20 and 45 days). Shannon's diversity index showed that the LAB supplementation of the HFD allowed increasing the diversity over time. Finally, the reduction of Simpson's index in HFD-CRL1446 and HFD-CRL431 showed a diversity gain over time.

**Table 3:** Alpha diversity metrics.

	**Experimental Groups**	Alpha diversity metrics^*^				
		**Sobs**	**Chao**	**Ace**	**Shannon**	**Simpson**
	SD	1400.00	8550.04	17653.82	3.48	0.20
20 d	HFD−20 d	1165.00	9516.07	16807.21	3.46	0.08
	HFD-CRL1446-20d	1288.00	11718.68	27741.89	3.15	0.15
	HDD-CRL1434-20d	1344.00	9621.51	25823.69	3.95	0.06
	HFD-CRL431-20 d	1250.00	12076.40	29261.24	3.25	0.11
45 d	HFD−20 d	1111.00	7788.68	16101.98	3.62	0.07
	HFD-CRL1446-45d	1464.00	10134.00	27230.89	4.22	0.06
	HDD-CRL1434-45 d	1421.00	12393.97	33202.88	4.01	0.06
	HFD-CRL431 – 45 d	1399.00	9862.09	25947.74	4.34	0.04

* *Sobs, Chao, Shannon, Ace, Simpsom's diversity of the intestinal fecal content of mice after the dietary intervention with a high-fat diet (HFD) with and without administration of CRL1446 (HFD-CRL1446), CRL1434 (HFD-CRL1434), and CRL431 (HFD-CRL431) at a dose of 1 x 10^8^ CFU/mL*.

**Figure 4:**
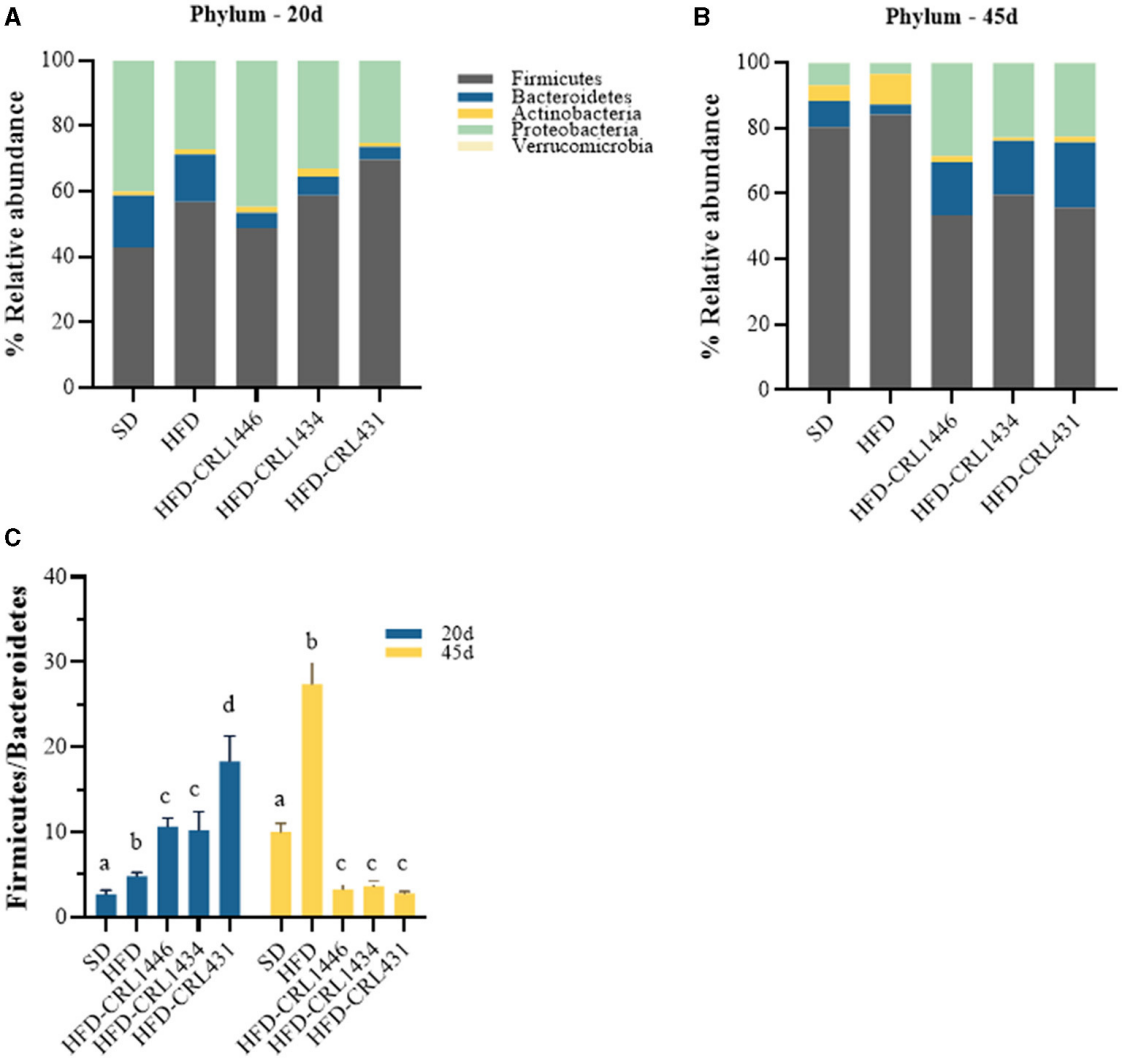
Relative abundance (%) of the *phila* **(A,B)** and F/B index (Firmicutes /Bacteroidetes ratio) **(C)** in the intestinal fecal content at 20 and 45 days of standard diet (SD) or a high-fat diet (HFD) administration with and without supplementation of CRL1446 (HFD-CRL1446), CRL1434 (HFD-CRL1434), and CRL431 (HFD-CRL431) at a dose of 1 x 10^8^ CFU/mL. Controls mice were fed with SD or HFD without bacterial supplementation. The microbial communities present in the mice fecal samples of the different groups were analyzed by massive and parallel sequencing of 16S rDNA amplicons. Each data group represents the average of 6 mice, and the 21 main genera that represent more than 99% of the intestinal microbiota are presented.

**Figure 5:**
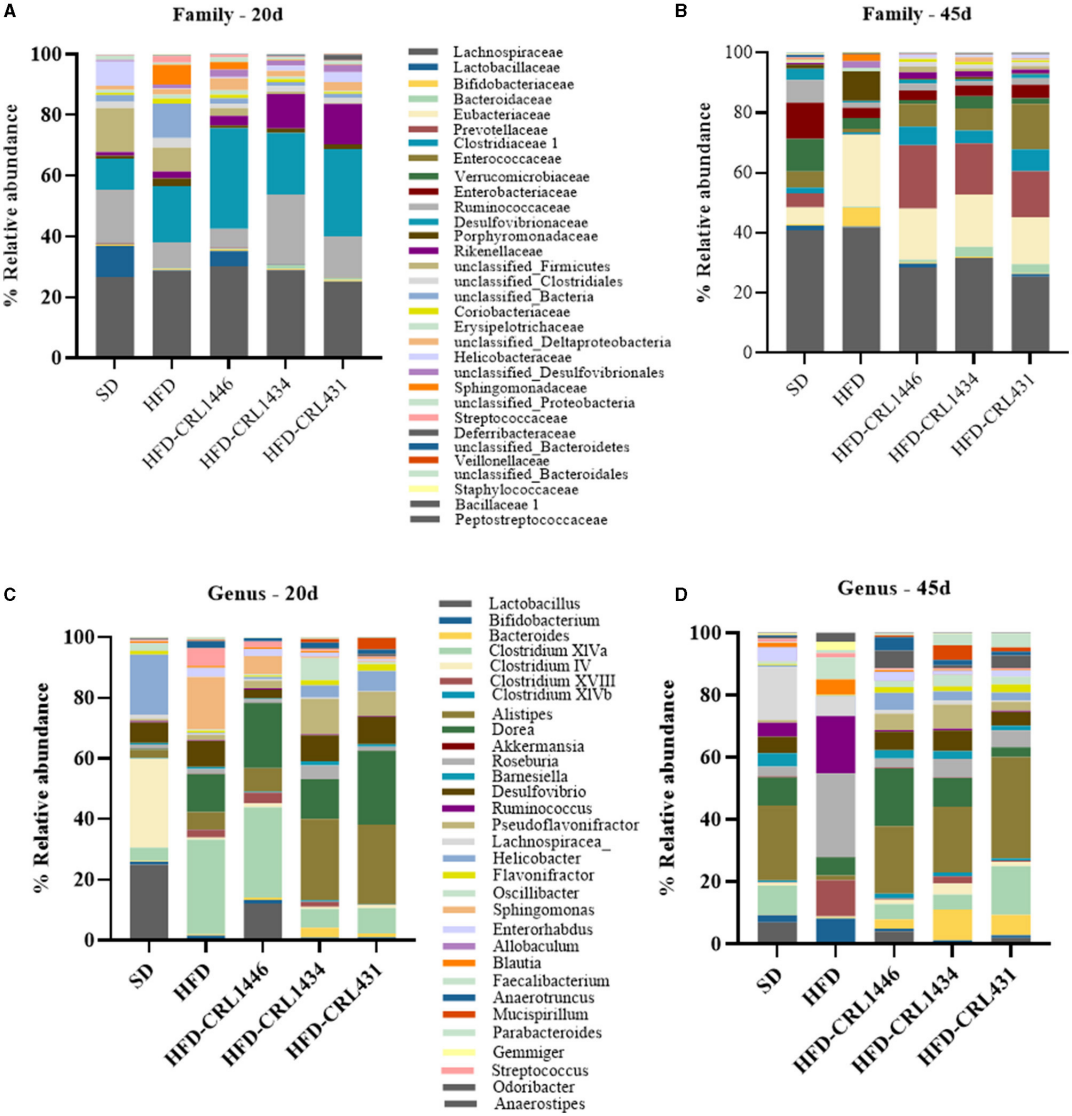
Relative abundance (%) *Family* **(A,B)**, and *genera* **(C,D)** in the intestinal fecal content at 20 and 45 days of standard diet (SD) or a high-fat diet (HFD) administration with and without supplementation of CRL1446 (HFD-CRL1446), CRL1434 (HFD-CRL1434), and CRL431 (HFD-CRL431) at a dose of 1 x 10^8^ CFU/mL. Controls mice were fed with SD or HFD without bacterial supplementation. The microbial communities present in the mice fecal samples of the different groups were analyzed by massive and parallel sequencing of 16S rDNA amplicons. Each data group represents the average of six mice, and the 21 main genera that represent more than 99% of the intestinal microbiota are presented.

Our results demonstrate essential changes in the intestinal microbiota composition at the Phylum (Figures 4A,B), Family (Figures 5A,B), and Genus (Figures 5C,D) level in experimental groups and the different treatment periods (20 and 45 d). The HFD group had a higher relative abundance of Firmicutes and a lower Bacteroidetes compared to SD group. At 45 days after LAB administration, we observed a decline of the Actinobacteria and Firmicutes phylum and increased Bacteroidetes and Proteobacteria (Figures 4A,B). In addition, the HFD group present the highest F/B index values compared with other study groups at 45 days, while the three studied strains favor a significant decrease in this index (Figure 4C).

At the end of the treatment period (45 d), the relative abundance on the Family level showed that LAB supplementation induces a higher abundance of Bacteroidaceae, Eubacteriaceae, Prevotellaceae, Clostridaceae, and Enterococacceae compared with 20 d (Figures 5A,B).

At the Genus level, differences between strains administration were observed. The LAB administrations increased *Lactobacillus* genus, principally with CRL1446 (Figures 5C,D). The *Bifidobacterium* genus increases at 45 days of treatment in the HFD group, but strains administration decreased this genus. The administration of LAB increased mainly the genera *Bacteroides* (principally with CRL1446)*, Clostridium* XIVa (principally with CRL431), *Alistipes, Dorea* (principally with CRL1446)*, Barnesiella* and, *Desulfovibrio Pseudoflavonifractor*. In contrast, the strains induced a decrease of *Roseburia, Ruminococuss, Clostridium* XVIII genus (Figure 5D).

**Figure 6:**
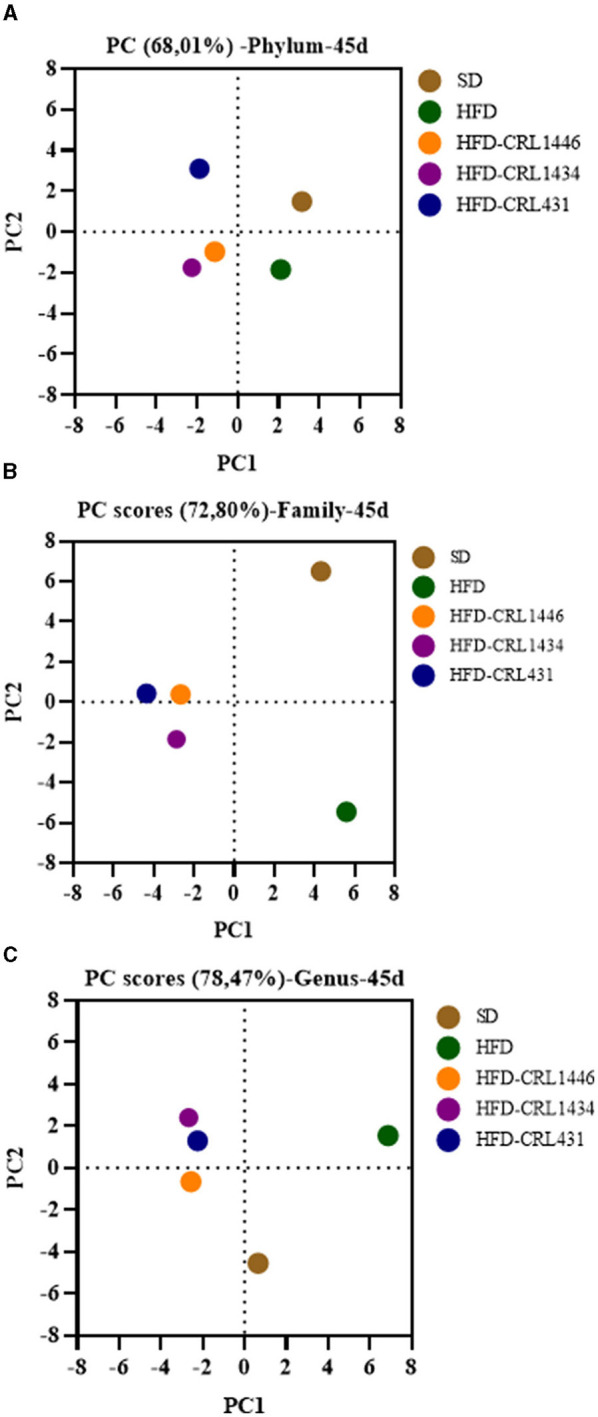
Principal component analysis of **(A)** Phylum, **(B)** Family, and **(C)** Genus of gut microbiota at 45 days of mice with administration of standard diet (SD) or a high-fat diet (HFD) with and without supplementation of CRL1446 (HFD-CRL1446), CRL1434 (HFD-CRL1434), and CRL431 (HFD-CRL431) at a dose of 1 x 10^8^ CFU/mL. Controls mice were fed with SD or HFD without bacterial supplementation.

The principal component analysis (PCA) was assayed to investigate the difference in microbial structure among different groups (Figures 6A–C). The HFD and SD groups showed a microbiota composition independently of each other. Moreover, the LAB groups had a separated cluster in comparison with the HFD and SD groups.

## Discussion

There is considerable evidence in pre-clinical and clinical interventions that demonstrates the use of probiotics as an alternative to improve the treatment and prevention of obesity-related disorders (10, 23). The different anti-obesity effects of traditional or not traditional probiotics strains are needed to be well-defined since its benefits are strains and/or dose-dependent (24). It is crucial to understand the strain-specificity and mechanisms involved in probiotics.

In the present pre-clinical study, we compared the effects of an HFD and the primary intervention with different adipomodulatory LAB; CRL1446, CRL1434, and CRL431 on immune-metabolic parameters and gut microbiota composition in obese mice. To establish the effects of time, we performed this work at 20 and 45 days. The administration of HFD for 45 days induced an increase in body weight, and it was associated with a marked increase of the feed conversion ratio (FCR) compared to the SD. The CRL431 supplementation showed no significant differences in the BWG. Similarly, Nuñez et al., in a 60-day obesity model, reported an increase in BWG induced by CRL431 (25). Andersson et al. also reported a body weight gain in the obese group supplemented with the probiotic *Lactiplantibacillus plantarum* DSM 15313 (26). In contrast, CRL1446 and CRL1434 strains showed BWG and FCR effects, inducing a significant decrease in both nutritional parameters. Many studies conducted in obese animals demonstrated decreased BWG, food consumption, or fat accumulation after probiotic supplementation (27–30). Also, in a previous study, in a 98-days metabolic syndrome model in mice, CRL1446 strain maintained its anti-obesogenic effect (14).

The available information suggests that the studied LAB had a strain-dependent effect on metabolism. The strains could induce modifications to the energetic metabolism and/or modulate the energy extraction capacity in the host.

Analyzing the adipose tissue in the HFD group, we showed hypertrophy and tissue weight gain (mesenteric, and epididymal) compared to the SD group. In epididymal adipose tissue, we observed an increase in the number of large adipocytes induced by HFD. It is established that the increase in adipose tissue size is associated with changes in the adipokine profile and metabolic dysregulation, which contributes to the development of chronic diseases linked to diets, such as obesity, type 2 diabetes, and cancer (7, 31). In our study, the HFD-induced hypertrophy of adipose tissue was correlated with increases in the production of plasmatic leptin (adipokine), IL-6 and TNF-α cytokines, and chemokine MCP-1. These pro-inflammatory molecules could be one mechanism by which the HFD promotes the immuno-metabolic imbalances associated with obesity (7). The strains CRL1446 and CRL1434 reversed the immune deregulation caused by the HFD across the gut and peripheral tissues (adipokine production by adipose tissue).

These strains completely arrest the adipose tissue hypertrophy, with a reduction in tissue weight and the area of the adipocytes, compared with the HFD group. Besides, it was associated with a significant reduction in plasma leptin levels, a key adipokine in the development of diet-induced obesity (32, 33). The state of hyperleptinemia observed in obesity was already reported by several authors, who also demonstrated the ability of certain macronutrients in the diet (lipids specifically) to induce leptin resistance (10, 11). Previously, we demonstrated the efficacy of microcapsules containing CRL1446 strain and *Lactobacillus johnsonii* CRL1231 to reduce in obese mice the plasma leptin levels, accompanied by the improvement of metabolic parameters and reduction of BWG, adiposity index, and fat deposition in the liver (15).

The CRL1446 and CRL1434 strains induced a reduction in leptin levels, reaching levels similar to the SD control. Reduction in leptin levels was correlated with decreased BWG, feed conversion ratio, plasma glucose, lipid levels, and a reduction in adipose tissue size. We showed that the *in vivo* adipose-modulatory effects correlated with previous *in vitro* results obtained with these strains (13). On the other hand, the supplementation with the CRL431 strain induced an increase in leptin levels compared to the HFD group. Moreover, we have reported an increase in leptin levels induced by this strain in a caloric-restriction model (16).

The chronic inflammatory state, characteristic of obesity, was observed when we analyzed plasma cytokine levels. We detected increases in the pro-inflammatory cytokines; TNF-α and IL-6, the chemokine MCP-1, and the reduction of IL-10 (a regulatory cytokine). Other authors also reported this pro-inflammatory state induced by HFD in both animals and humans, and a link was established between the development of this pro-inflammatory state and insulin resistance in type-diabetes II (11, 34). In our study, the strains with the capacity to lower leptin levels also reduced the pro-inflammatory molecules (TNF-α, IL-6, and MCP-1). This effect was also associated with the increase of IL-10 cytokine. So, we demonstrated the immunomodulatory capacity of CRL1446 and CRL1434, which maintained IL-10 levels next to the values of the SD group and reduced leptin, TNF-α, IL-6, and MCP-1 levels. While the supplementation with the CRL431 strain, despite increasing IL-10 levels, could not reverse the increases in pro-inflammatory cytokines induced by HFD. These results could be due to the higher leptin levels associated with the CRL431 strain.

In this context, it was reported by several authors a negative relationship between obesity and IL-10 levels (35, 36); in some cases, this cytokine correlated positively with energy homeostasis and negatively with insulin resistance. Recently, Nakata et al. reported that the overexpression of IL-10 in leptin-deficient mice reduced hyperphagia, adiposity, glucose intolerance, and insulin resistance and attenuated TNF-α expression (37). Furthermore, a clinical trial in women with polycystic ovary syndrome, a disease that also has features of the metabolic syndrome, including insulin resistance, obesity, and a pro-inflammatory estate, demonstrated that *Lactobacillus* supplementation could reduce the inflammation through IL-6, CRP reduction, and increase of IL-10, in congruence with a decrease in weight and body mass index (36). Thus, we suggest that increases in IL-10 associated with reduced leptin levels induced by strains CRL1446 and CRL1434 may be the primary mechanisms by which they exert their antiobesogenic effects.

It is considered that IL-6, together with TNF-α, could play a key role in developing insulin resistance and pathologies related to obesity (7). About 35% of circulating IL-6 is produced by adipose tissue, and it increases with the expansion of this tissue. Besides, leptin also correlates with many obesity parameters, and its secretion also increases with the enlargement of adipose tissue (7). This peptide acts promoting the proliferation of pro-inflammatory cells and cytokines, like TNF-α and IL-6. The immunomodulatory probiotic CRL431 previously showed to raise leptin levels in mice under a caloric restriction diet (16). In general, it is expected that positive impacts of probiotics on obesity and associated metabolic disorders are related to a decrease in serum leptin values or leptin expression (10, 38, 39). However, numerous animal and clinical studies showed discrepancies concerning the effects of probiotics on obesity and their impact on serum leptin levels or leptin expression (40, 41). Further, a strain-specific effect on leptin was proposed for probiotics (10). In this study, the supplementation with the CRL431 strain did not improve weight or adipose tissue compared to HFD control, which could relate to the maintenance of resistance to leptin and the increase in IL-6 levels. In addition, these results could be associated with the duration of the treatment (45 d). A previous work using the same strain for 60 d of administration in a different obesity mice model showed decreased inflammation markers (42).

Elevated cholesterol levels are commonly associated with dyslipidemia in diet-induced mice obesity models (10, 11, 15). We observed that the HFD increased total cholesterol levels, and LAB supplementation reduced this lipid in plasma, suggesting a hypocholesterolemic effect. The CRL1446 and CRL1434 strains showed higher hypocholesterolemic effects than CRL431. Several studies conducted in obese animals have demonstrated the positive influence of various *Lactobacillus* strains on cholesterol metabolism (43–45). Besides, we previously reported a hypocholesterolemic effect of strain CRL1446 on a murine caloric restriction model and in mice fed with SD supplemented with CRL1446-cheese (46, 47). Also, in previous work, we proved the efficiency of oral administration of spray-dried microcapsules containing feruloyl esterase producing CRL1446 and *L. johnsonii* CRL1231 to improve metabolic parameters (including lipids profile) and atherogenic index (15). Consequently, we showed the ability of CRL1446 strain to reduce cholesterol levels independently of the diet administered or the matrix used to carry the probiotic in an obesity mice model. Also, Bhathena et al. reported that the feruloyl esterase producing *Limosilactobacillus fermentum* LF11976 decreased serum lipids in hypercholesterolemic hamsters (48). Feruloyl esterases are enzymes responsible for cleaving insoluble wall-bound hydroxycinnamates, releasing ferulic acid (among other hydroxycinnamic acids) from plant matrixes (49). Ferulic acid can inhibit hydroxymethyl-glutaryl CoA reductase, a rate-limiting enzyme in the biosynthesis of cholesterol, and cholesterol acyltransferase, an enzyme that esterifies cholesterol in tissues, mainly in the liver. Also, ferulic acid may improve the excretion of acid sterols (50). These biological activities explain the hypocholesterolemic properties of ferulic acid. Previously, it has been shown that the CRL1446 strain has *in vivo* and *in vitro* high feruloyl esterase activity (51). Therefore, we suggest that feruloyl esterase activity observed in this bacterium could be implicated in its hypocholesterolemic effect.

On the other hand, other authors have reported the capacity of the intestinal microbiota to influence the metabolism of cholesterol in both animals and humans (52). In our study, LAB partly re-establishes the symbiosis state and could indirectly affect cholesterol levels. CRL1434 strain is an immunomodulatory strain that had not previously been evaluated in obesity mice models, and it also showed a hypocholesterolemic effect (13). This strain showed the ability to synthesize conjugated linoleic acid (CLA), which could be associated with its capacity to reduce plasma cholesterol levels (53). The administration of bacteria able to produce CLA, a molecule with vast biological functions, including anti-obesity properties and improvement of the lipids profile, at the intestinal level is essential for human health. Hence, this strain could be used to develop functional dairy products with high concentrations of conjugated fatty acids or as a probiotic to promote the synthesis of these bioactive lipids in the intestine (53).

Scientific evidence has demonstrated the strong relationship between gut microbiota, inflammation, and metabolic disturbances related to obesity and insulin resistance (4, 7). Obesogenic diets are considered a determinant factor that modulates intestinal microbiota and is responsible for developing metabolic inflammation and energy metabolism imbalance (54). The gut microbiota composition and diversity can change in the short term of HFD consumption (45). Using high throughput sequencing methods to study intestinal microbiota changes, we observed that it was profoundly affected by the HFD, which induces an increase in the F/B index compared to the SD. The rise of this index is due to an increase in Firmicutes members, which is in agreement with other studies in rodents and humans (55). However, controversial evidence regarding the two dominant phyla (Firmicutes and Bacteroidetes) in obesity are documented and established inverse associations between these bacterial taxa (56, 57).

Cani *et al*. reported that intestinal dysbiosis and HFD induce increased permeability in the gut, promoting metabolic endotoxemia increasing the LPS from Gram-negative bacteria in the blood (58). The LPS favors the inflammatory response and triggers hyperglycemia and insulin resistance. This impact of diet on the gut microbiota composition offers new approaches in nutritional interventions through the manipulation of the intestinal ecology or introduction of specific beneficial microbial species. In this sense, specific probiotics can modulate the gut microbiota and prevent chronic low-grade inflammation and altered energy metabolism linked to obesity and metabolic syndrome (7).

BWG and FCR are also strongly associated with the gut microbiota composition, which determines the obtention of energy from the diet (59). Several authors correlated the Firmicutes phylum with BWG and the Bacteroidetes phylum with loss of weight. The F/B index is closely related to the energy extraction capacity of the diet by the host, and it has been established that the microbiota of obese individuals has a more remarkable ability to extract energy and deposit it in the form of body fat (60). The F/B index reduction in the obese mice with LAB-supplemented diets may partly explain the decrease in BWG.

Our results showed that HFD increased plasma glucose levels in comparison with SD. Diet supplementation with CRL1446, CRL1434, and CRL431 strains showed a reduction in glucose levels regarding the HFD group. The influence on glucose levels may be associated with gut microbiota changes promoted by LAB administration. Indeed, a marked Firmicutes reduction and the F/B index were observed after the LAB supplementation.

In this context, several authors have reported the influence of gut microbiota on insulin sensitivity and glucose tolerance and the decrease of pro-inflammatory cytokines such as TNF-α and IL-6 in plasma (45). The LAB effect on glucose metabolism could also be associated with their immunomodulatory profile. In effect, CRL1446 and CRL1434 strains induced the reduction of pro-inflammatory cytokines.

There is sufficient evidence relating the beneficial effects of probiotics on metabolism to the production of metabolically active compounds (through the biotransformation of complex polysaccharides, proteins, and polyphenols into short-chain fatty acids (SCFA), bioactive peptides, and phenolic compounds, respectively), or their ability to modulate the inflammatory response or gut microbiota composition, which reflects in the amelioration of chronic low-grade inflammation and oxidative stress linked to metabolic diseases (7, 61). In particular, SCFA (mainly acetate, propionate, and butyrate), which constitute the main products of dietary fiber fermentation in the intestines by microbiota, are often considered essential metabolites for probiotics exert their effects (62). SCFA are presumed to improve energy homeostasis and inflammation by activating eukaryotic cells receptors (free fatty acid receptors FFAR2 and FFAR3, and G-protein-coupled receptor GPR109A) or inhibiting histone deacetylases (62, 63). Thus, SCFA performs its effects at different levels. By decreasing inflammation (e.g., by modulating the secretion of interleukins, inducing T regulatory cells, interfering with the maturation of monocyte-derived dendritic cells, and down-regulating TLR4 receptor in macrophages); increasing the secretion by colonocytes of glucagon-like peptide-1 (GLP-1) and peptide YY (PYY) that regulates food intake and improves β-cell function, and GLP-2 that also regulates food ingestion and improves intestinal barrier integrity and intestinal function; and increasing insulin sensitivity in muscle (62, 63). In fact, in previous work, we demonstrated that CRL1446 strain administration to a mice model of metabolic syndrome improved metabolism, and this effect was associated, among others, with the modulation of gut microbiota and the increase in the concentration of 3-hydroxyphenylpropionic and butyric acids in colonic content (14).

It is known that diet is a major factor in modulating the intestinal microbiota inducing environmental modifications (pH, bile acids, SCFA) in the gut ecosystem. In addition, long-term and short-term dietary patterns (HFD and HFD-probiotics) have been shown to induce gut microbiota structure and function modifications (64). Changes in microbiota composition in HFD are probably related to these modifications in the environmental conditions of the gut. The increase in the fat percentages to the detriment of fiber in the diet can expedite the growth of some species of bacteria but negatively impact other species. For instance, reduction in fiber percentage in the diet impairs fiber-fermenting bacteria that results in changes in pH and SCFA content, as well higher fat proportion stimulate bile acids conjugation and increasing organic sulfur availability (64). Therefore, the availability of nutrients and the changes in the gut environmental conditions would trigger the shift in the microbiota diversity.

In our work, as expected from the literature, we have observed a dysbiotic microbiota in the intestine of mice fed HFD. This dysbiosis was characterized by an increased relative abundance of Firmicutes and a decrease of Bacteroidetes. Ley et al. showed that after dietetic treatment in humans, the relative abundance of the main phyla is reversed (65). In this study, at the end of LAB administration, an effect similar to weight loss interventions was observed. In this regard, it should be noted that recent studies indicated that the associations established between the abundance of Bacteroidetes and Firmicutes phylum and HFD are oversimplifications. The link between metabolism and microbiota composition is higher sophisticated, and the genus diversity and the species level are pivotal in the obesity context (66).

Lachnospiraceae family is conserved in HFD groups. Some members of Lachnospiraceae are among the leading producers of SCFA, but different taxa of Lachnospiraceae are also associated with various diseases (67). Despite LAB supplementation to HFD mice induced a decrease in Lachnospiraceae family, the relative abundance of *Dorea* genus (one of the principal genera within this family and producer of SCFA) is increased. *Dorea* genus was positively affected by CRL1446 and CRL 1434 administration, which could favor the SCFA production.

It was also found that the Prevotellaceae family was highly present in the mice with LAB administration. However, we have not detected an abundance of *Prevotella* genus. It is known that an increase in the *Prevotella* genus facilitates the fermentation of the carbohydrates and increases the production of SCFA (68).

In terms of gender analysis, we also observed that the HFD promoted the abundance of *Bifidobacterium, Ruminococcus*, and *Clostridium* XVIII. Some species, such as *Ruminococcus bromii, Ruminococcus obeum* have been associated with HFD and the development of obesity (68). Previous studies have associated *Clostridium* XVIII with abnormal lipid profiles, gastrointestinal disorders, and colonic inflammation in HFD fed rats. In addition, *Roseburia* genera were related to abnormal lipids parameters (68). Further studies related to lipid metabolism and microbiota are necessary to clarify these issues.

Our results showed that the HFD group with LAB supplementation caused an increase in the relative abundance of *Alistipes, Barnesiella* (within Bacteroidetes phylum), *Pseudoflavonifractor, Clostridium* XIVa (within Firmicutes phylum)*, Desulfovibrio* (within Proteobacteria phylum). Hu et al. showed negative correlations between lipid metabolism-associated abnormal parameters in HFD rats and the relative abundance of *Alistipes* genus, suggesting a beneficial effect of this bacteria (68). Jiang et al. reported an increase in the relative abundance of genera *Alistipes* and *Prevotella* in healthy lean patients compared to individuals with non-alcoholic fatty liver disease (69). Louis *et al*. showed that obese patients who had success in weight loss in 2 years, at the beginning of the study, had a gut microbiota enriched in *Alistipes* and *Pseudoflavonifractor* compared with patients who were less successful in reducing weight (70). The supplementation with LAB, particularly CRL1446 strain, enriches the relative abundance of the *Lactobacillus* genus. This genus correlates with beneficial effects on the host and positively with life expectancy (71).

The supplementation with probiotic LAB can improve the relative abundance of certain beneficial bacteria genera on the intestinal microbiota of HFD fed mice through diverse mechanisms (72). These mechanisms may include limiting the growth of undesired microorganisms by the production of metabolites with antimicrobial or inhibitory properties (i.e., bacteriocins, lactic acid), competition for dietary substrates, the competitive exclusion for binding sites or receptors on the intestinal mucosa, stimulation of innate immune response (host's production of β-defensin and IgA) (72). In addition, probiotic LAB can raise the abundance of beneficial bacteria genera through the production of growth substrates (exopolysaccharides, vitamins, or SCFA) for other bacteria, by altering the dynamics of carbohydrate utilization, the improvement of the intestinal barrier function or reduction of inflammation that may result in maintenance of immune tolerance favoring the colonization and persistence in the intestinal lumen of certain bacteria (72).

According to the obtained results, we suggest that LAB supplementation to the HFD group could counteract the detrimental effects induced by HFD on gut microbiota, favoring an enrichment of genera that correlated positively with healthy lean individuals. Thus, we support the hypothesis that the same diet may impact differently according to the basal composition of the intestinal microbiota.

### Conclusions

This study allowed to establish the effect of diet, time of treatment, and specific LAB supplementation on the gut microbiota composition and metabolic-immunological parameters in obesity disease. LAB supplementation in a HFD mice model can ameliorate the effects exerted by the HFD, improving body weight and metabolic parameters in a strain-dependent manner. These effects can be associated with intestinal microbiota, adipose tissue, adipokines, and cytokine profile changes.

Specifically, the oral administration of CRL1446 and CRL1434 strains is related to the reduction in the leptin levels and pro-inflammatory cytokines and was associated with greater lipid-lowering and hypoglycaemic effects. In contrast, the CRL431 strain increased leptin level and did not change the cytokines profiles regarding the HFD group. Thus, the functional properties of the CRL1446 and CRL1434 strain can support their use as a complement for the prevention or primary intervention of metabolic disorders associated with obesity. The possibility of using specific probiotic strains as a strategy to minimize or improve changes induced by the diet arises as an attractive and viable alternative for attenuate the adverse impact of obesity on metabolic health.

## Data Availability Statement

The datasets presented in this study can be found in online repositories. The names of the repository/repositories and accession number(s) can be found below: NCBI SRA BioProject, accession no: PRJNA749164.

## Ethics Statement

The study protocol was approved by the Ethical Committee of Laboratory Animals Center of CERELA (approval number CRL-BIOT- EF-2012 / 2A). Written informed consent was obtained from the owners for the participation of their animals in this study.

## Author Contributions

EF and PG-C: conceived and designed the experiments. EF, AM, RR, and MR: performed the experiments. EF, RR, and PG-C: analyzed the data. CF and EP: deposited raw sequence data in a public repository. PG-C, ST, and RM: wrote draft and the final version of the manuscript. All authors contributed to the article and approved the submitted version.

## Funding

This study was supported by grants: - PIP 215 from CONICET, Argentina. - BID-PICT 2017-3975, BID-PICT 2018-2151 from FONCyT.

## Conflict of Interest

The authors declare that the research was conducted in the absence of any commercial or financial relationships that could be construed as a potential conflict of interest.

## Publisher's Note

All claims expressed in this article are solely those of the authors and do not necessarily represent those of their affiliated organizations, or those of the publisher, the editors and the reviewers. Any product that may be evaluated in this article, or claim that may be made by its manufacturer, is not guaranteed or endorsed by the publisher.
